# Reaping the Benefits of Microorganisms in Cropping Systems: Is the Regulatory Policy Adequate?

**DOI:** 10.3390/microorganisms9071437

**Published:** 2021-07-02

**Authors:** Ingvar Sundh, Teresa Del Giudice, Luigi Cembalo

**Affiliations:** 1 Department of Molecular Sciences, Swedish University of Agricultural Sciences, P.O. Box 7015, 750 07 Uppsala, Sweden; 2 Department of Agricultural Sciences, University of Naples Federico II, 80055 Portici, Italy; Teresa.delgiudice@unina.it (T.D.G.); cembalo@unina.it (L.C.)

**Keywords:** plant-beneficial microorganisms, regulatory framework, legislation, biological control, biocontrol, plant protection product, plant growth promotion, biostimulant, biofertilizer, microbial safety assessment

## Abstract

Within food plant cropping systems, microorganisms provide vital functions and ecosystem services, such as biological pest and disease control, promotion of plant growth and crop quality, and biodegradation of organic matter and pollutants. The beneficial effects of microorganisms can be achieved and/or enhanced by agricultural management measures that target the resident microbial biodiversity or by augmentation with domesticated and propagated microbial strains. This study presents a critical review of the current legislation and regulatory policies pertaining to the utilization of plant-beneficial microorganisms in the European Union (EU). For augmentative approaches, the nature of the intended effect and the product claim determine how a microbiological product is categorized and regulated, and pre-market authorization may be mandatory. Typically, microbial products have been incorporated into frameworks that were designed for evaluating non-living substances, and are therefore not well suited to the specific properties of live microorganisms. We suggest that regulatory harmonization across the sector could stimulate technical development and facilitate implementation of crop management methods employing microorganisms. Possible scenarios for regulatory reform in the longer term are discussed, but more investigation into their feasibility is needed. The findings of this study should serve as a catalyst for more efficient future use of plant-beneficial microorganisms, to the benefit of agriculture as well as the environment.

## 1. Introduction

Many of the activities and functions of microorganisms—and other beneficial organisms—within agricultural settings are vital for good plant crop development. These functions and the corresponding provider organisms are often referred to as ‘ecosystem services’ and ‘ecosystem service providers’, respectively [1,2]. Genetic resources and ‘cultural’ services (e.g., spiritual enrichment, recreation, and aesthetic experiences) provided by biodiversity are also included as ecosystem services.

When the factors and conditions that control a particular beneficial microbiological function are known, that function can potentially be stimulated through targeted management measures. Alternatively, beneficial microorganisms can be applied to the cropping system, either as consortia in, e.g., organic fertilizers or soil conditioners, or as isolated and propagated single microbial strains. The latter approach is used most widely for the microbial control of agricultural pests [3,4] (the word ‘pests’ is used in a broad sense, including invertebrate pests, plant pathogens, and weeds) and microbial stimulation of plant growth or health by mechanisms other than pest control, i.e., ‘biostimulation’ [5].

**Figure 1:**
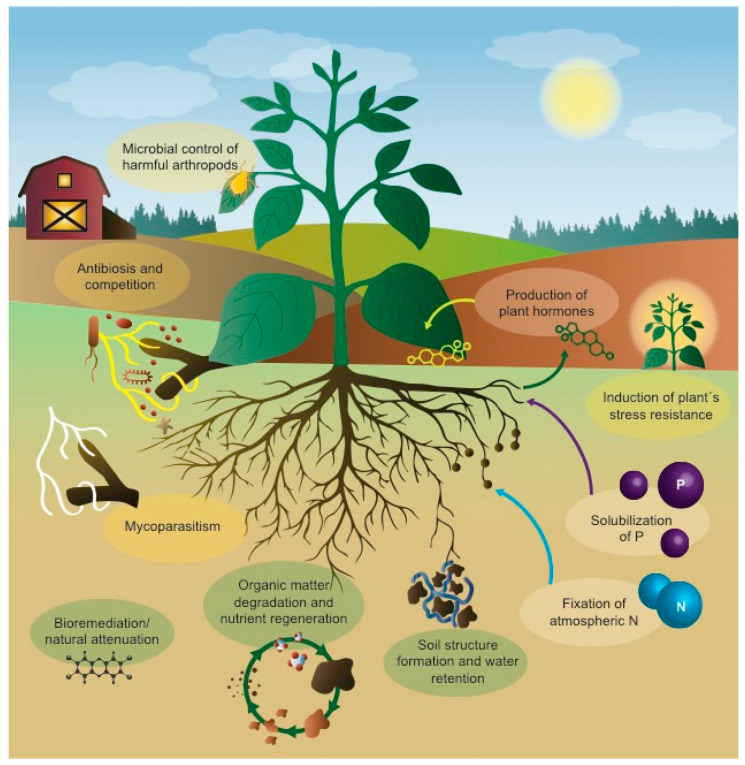
Graphical overview of plant-beneficial functions and ecosystem services provided by microorganisms in cropping systems. Functions and services can be grouped broadly into pest and disease control (shown mainly on the left-hand side), stimulation of plant growth and crop quality (mainly on the right-hand side) and biodegradation/soil formation (the soil compartment).

Figure 1 presents an overview of the plant-beneficial activities of microorganisms in cropping systems. The application of microbial control agents (MCAs) for the biological control of plant pests is often referred to as an ‘alternative’ approach to chemical pesticides, but microbial control is not a new technology. The first field trials involving augmentative biological control with a microorganism were performed in the Ukraine in the 1880s with the insect pathogen *Metarhizium anisopliae* [6], and the first microbial products for pest control (of harmful insects) were available commercially as early as the 1920s, if not earlier, before the advent of synthetic organo-chemical pesticides (see historical overviews by Lord [7], Ravensberg [8], and Sundh and Goettel [9]). Besides control of pest insects by entomopathogens [4], various antagonistic and mycoparasitic microorganisms have found wide use in the control of plant pathogens [3]. Additionally, some phytopathogens have been used in biological control of invasive and agricultural weeds (recently reviewed by Morin [10]).

‘Plant biostimulants’ include various types of substances or microorganisms that can enhance plant growth or crop quality [5,11]. They are applied within cropping systems, but they do not comply with typical definitions of either pesticides or fertilizers. A plant biostimulant has been defined as ‘any substance or microorganism applied to plants with the aim to enhance nutrition efficiency, abiotic stress tolerance, and/or crop quality traits, regardless of its nutrient content.’ [12]. Industrial development and marketing of microbial biostimulants (MBSs) first took place in the 1920s, when nitrogen-fixing (diazotrophic) root nodule bacteria were distributed for seed inoculation [13]. Since then, diazotrophic bacteria have been widely used in legume cultivation. Other mechanisms of MBSs are: (i) facilitation of solubilization, availability, and/or plant uptake of nutrients; (ii) production of phytohormones that promote plant growth processes; and (iii) production of substances that can ameliorate abiotic stress in plants (e.g., drought or salinity stress) [11].

The metabolic capabilities of microorganisms make them potentially useful for augmentative bioremediation of agricultural soils contaminated with heavy metals or organic pollutants, e.g., chemical pesticides [14,15]. Additionally, an improved understanding of the capacity of some fungi and bacteria to degrade mycotoxins (reviewed by Taheur et al. [16]) has provoked interest in applying microorganisms to crop produce for detoxification purposes [17].

In order to ensure an acceptable level of human and environmental safety when using an organism(s), some interventions utilizing microorganisms are regulated. As well as verifying safety, regulatory measures can ensure that microbiological products are efficacious, appropriate quality control is undertaken, and intellectual property is protected. Regulatory provisions for the use of microorganisms vary considerably across application areas, but in several risk assessment and authorization of the organism and/or the corresponding product is mandatory [18,19]. For example, in the European Union (EU), authorization of microbial products for the control of plant pests is harmonized by common EU legislation, whereas microbial biostimulants are currently only under national governance. Disparate and unclear regulatory demands are a cause for concern, because they can suppress economic incentives for investment in the development of new methods and products [20].

To assess the potential risks of a microbiological application, both (i) the fundamental biological properties and any corresponding hazards of the particular strain(s), and (ii) the nature and claimed effect of the intervention, should be evaluated. Thus, regulatory frameworks for evaluating microorganisms need to strike a balance between harmonization based on the shared fundamental properties and hazards of groups of microorganisms, vs. diversification motivated by differences in proposed use (and thus exposure) and desired effect. Additionally, regulatory provisions can be expected to be proportional and motivated [21] and accord with the actual risks to a reasonable level. Exaggerated demands can lead to unnecessarily complicated and prolonged evaluation processes, and thus to the inefficient use of common resources. In contrast, a policy that is too permissive can lead to unacceptable human or environmental risks.

In this study, we critically review the various procedures and information requirements of current regulatory frameworks and policies that can affect the utilization of plant-beneficial microorganisms in crop production, with an emphasis on the situation in the EU. Special attention is given to frameworks that cover microbial plant protection products (PPPs) and microbial biostimulants, respectively. We start by considering the features of the current regulatory frameworks within different application sectors, and go on to discuss whether the regulatory heterogeneity is motivated by the divergent hazards and risks of microorganisms. We then highlight current inconsistencies, and propose scenarios for the reformation and harmonization of current legislation, with a view to simpler evaluations and product authorizations in the future. Our conclusions are intended for regulatory institutions, policy makers, industry, and interest organizations within the sector, in order to increase utilization of microbial biodiversity in crop production while retaining an adequate level of human and environmental safety. For example, our insight could contribute to updates in the EU legislation regarding microbial PPPs within the Commission’s Farm to Fork initiative [22], or implementation of the new EU fertilizer regulations [23].

## 2. Current Regulations: Augmentation with Microorganisms

The main factors determining whether augmentation with a plant-beneficial microorganism(s) is regulated are the intended type of effect and the product claim.

### 2.1. Microbial Pest and Disease Control

The distinction between microbial pest control and biostimulation is not immediately obvious, but microorganisms that stimulate a plant’s tolerance to *biotic* stress (i.e., to pests) are categorized as PPPs, while those that stimulate a plant’s tolerance to *abiotic* stress are categorized as ‘plant biostimulants’ (see the overview of regulations in Table 1).

#### 2.1.1. The EU: Current Situation

Microorganisms for plant pest control fall under common EU regulations for PPPs [16]. The major features of the regulations are summarized below and in Table 1; for more details consult Frederiks and Wesseler [37]. PPP regulations are applicable whenever the claim is control of plant pests (including pre- as well as post-harvest applications), and pre-market approval of new microorganisms and authorization of the corresponding formulated products is mandatory.

**Figure 2:**
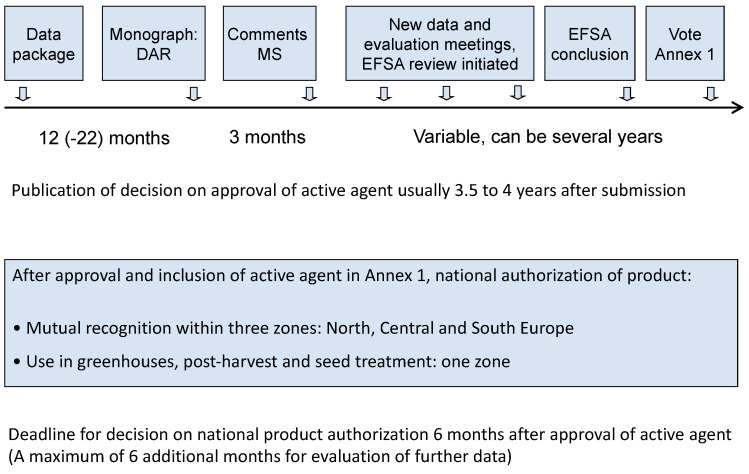
The EU authorization process for microbial biocontrol agents according to Regulation 1107/2009 for plant protection products. DAR = draft assessment report, MS = member states, EFSA = European Food Safety Authority.

The authorization process is complex (Figure 2) and the same formal requirements have to be fulfilled for microbial active agents and products as for chemicals; however, separate data requirements [25,26] and evaluation principles [27,28] apply to microbial PPPs. The re-publication of the data requirements and uniform principles for microorganisms in 2013 and 2011 were for legal and administrative purposes, and the legal documents from 2001 and 2005 were not updated in the process. In the Farm to Fork initiative [22], however, the EU Commission set out updates to the legislation regarding microbial PPPs to be applied within the next few years.

The current PPP regulations from 2009 stipulate that a new active agent and the corresponding products can be categorized as ‘low-risk’. The maximal time devoted for evaluating a low-risk PPP is shorter (but additional information can be requested) and approval should be for 15 instead of 10 years. The criterium for categorizing a microbial agent as low risk is that it does not carry transferable genes for resistance to antimicrobials of importance within human or veterinary medicine [38]. An EU Commission notice indicates that most microbial PPPs are expected to fulfill the criterium for low risk [39].

#### 2.1.2. Global Outlook

Similar to the EU, the regulatory systems for MCAs in non-EU countries are often aligned with the systems for regulating pesticides (see overviews in, e.g., [31,40,41]). The regulatory conditions within the EU have often been compared with those in the USA [37,40,42], and many studies over several decades have claimed that the EU framework, in particular, is unsuitable for microorganisms and has been a major obstacle to the market introduction of microbial pest control agents (e.g., [19,37,43,44,45,46,47,48,49,50,51,52,53]). Unlike the situation in the EU, evaluation of MCAs in the USA is performed by a special unit under the Environmental Protection Agency (EPA), with personnel dedicated to ‘biopesticides’, i.e., semiochemicals, plant extracts and any other substances of biological origin, as well as microorganisms.

### 2.2. Microbial Plant Biostimulation

#### 2.2.1. The EU: Current Situation

In contrast to the MCAs, MBSs are not subject to an EU-wide framework (Table 1). The situation ranges from free market access (Ireland, United Kingdom) to a pre-market authorization process similar to the PPP process (France, Hungary) [35,36,54]. Individual countries usually regulate biostimulants under the legislation for fertilizers. Definitions and terminology differ, and the regulations usually do not target ‘biostimulants’ per se, but various types of products within the fertilizer sector. This situation has hampered development of a common market for biostimulant products [12,55,56]. However, it is notable that, similar to the MCAs, MBSs are regulated within frameworks where the main scope is other, non-living, types of substances.

#### 2.2.2. The EU: Forthcoming New Regulations

The regulatory conditions for biostimulants in the EU will change with the implementation of harmonized new regulations for fertilizer products [23] that will come into force on 16 July 2022. Based on the various modes of action of biostimulants, the regulations consider biostimulants to be ‘by nature more similar to fertilizing products than to most categories of plant protection products’ ([23], introductory point 22). The new fertilizer regulations contain provisions for updating the PPP regulations with respect to the features that distinguish between microbial PPPs and biostimulants.

Product function category (PFC) no. 6 of the new regulations contains Plant Biostimulants and PFC 6A Microbial Plant Biostimulants. Each PFC is subject to specific safety and quality assurance requirements. Microorganisms complying with two criteria relating to drying methodology and taxonomy can be added to a positive list, obtain CE marking, and thus be made available on the EU market (see Appendix A for more details about the new regulations). For a new microbial biostimulant that does not fulfill both criteria, information related to the taxonomy and biology of the microorganism has to be submitted. The regulations state (introductory point 66) that if there are no harmonized standards, specifications are needed for requirements and tests to verify the conformity of a product with the CE system. Thus, for ‘new’ microorganisms not belonging to any of the four groups on the current positive list, there will be a case-by-case approach for certification according to standardized criteria, which need to be developed. Unlike the regulations for microbial PPPs, the new EU fertilizer legislation does not regulate the use of products, and pre-market authorization of a new MBS is not required.

#### 2.2.3. Global Outlook

World-wide, as within the EU, the regulatory conditions for plant biostimulants vary, and biostimulants often fall under legislation for fertilizers. Contributing to terminological confusion, MBSs are also commonly referred to as ‘biofertilizers’ [57,58]. The term ‘biofertilizer’ can refer exclusively to inoculated, live organisms such as N-fixating root nodule bacteria, mycorrhizal fungi, or nutrient-solubilizing bacteria (Government of India [59], Grand View Research [60]), or to non-microbial plant biostimulating substances as in regulations in Brazil [36]. Registration of MBSs is in many cases valid for no more than three to five years (Canada, South Africa, India, and Brazil). However, the time to authorization/registration can be limited to a few months. The information requirements concerning safety and effectiveness data for the MBS product also varies, with more extensive data required for authorization in Brazil and Canada than in South Africa, USA and India (see Traon et al. [35] and Caradonia et al. [36] for more details on regulatory conditions for MBSs outside Europe).

### 2.3. Biodegradation/Bioremediation

There is no EU-wide regulatory framework for applications of wild-type microorganisms to enhance biodegradation of pollutants or toxins, and we have not found any proposals or evidence that such regulations would be motivated on scientific or any other grounds. However, national legislation concerning biotechnology or environmental protection may be applicable, as, for instance, in Sweden (Table 1).

### 2.4. Convention on Biological Diversity: The Nagoya Protocol

The United Nations Convention on Biological Diversity (CBD) has three main objectives: (i) the conservation of biological diversity; (ii) sustainable use of components of biological diversity; and (iii) fair and equitable sharing of any benefits that arise from the utilization of genetic resources. Utilization includes research and development as well as any subsequent practical applications and commercialization. The CBD defines genetic material as ‘any material of plant, animal, microbial or other origin containing functional units of heredity’ and genetic resources as ‘genetic material of actual or potential value’ (https://www.cbd.int/convention/; accessed on 2 July 2021). The convention addresses proprietary rights but not questions relating to human or environmental safety. In the EU, there are regulations [61] and guidance [62] that introduce measures for compliance with the Nagoya protocol.

The CBD states that all countries have sovereign rights to their genetic resources and that benefits arising in a receiver country should be shared with the provider country in a fair and equitable way. Benefits arising from the use of genetic resources can be monetary as well as non-monetary. The Nagoya protocol, a supplement to the CBD, sets out procedures for access and subsequent benefit-sharing. In bilateral agreements, access, utilization, and benefit-sharing are regulated by primary informed consent (PIC) and mutually agreed terms (MATs).

Although there can be no doubt that the intentions of the convention are commendable, the CBD and Nagoya protocol can have the unintended consequence of hindering general access to biological materials, and therefore the potential for carrying out basic microbiological research and development [63,64]. For at least 10 years, scientists have stressed that any limitation on the exchange of biological materials can be a threat to biological pest control [65,66]. The International Organization for Biological Control (IOBC) has developed standards for best practice for biological control practitioners to comply with the Nagoya protocol [67]. These standards were developed specifically for invertebrate biological control agents, but the IOBC aims to develop best practice standards for microorganisms as well (Peter Mason, in e-mail 16 October 2020).

EU regulations for the Nagoya protocol were published in 2014, but implementation within EU countries is still underway [68]. Thus, the consequences of the Nagoya protocol for the future development, production, and commercial use of plant-beneficial microorganisms in the EU cannot yet be ascertained.

## 3. Current Regulations: Stimulation of Resident Microorganisms

### 3.1. Conservation Biological Control

Agricultural systems harbor numerous microorganisms that repress pest populations without any human intervention (see e.g., [3,69,70]), a phenomenon referred to as ‘natural biological control’ [71,72]. In conservation biological control, active management aims to enhance the numbers and/or activity of such resident, pest-controlling organisms. This is a well-established method in the area of beneficial arthropods, but has been less well studied for microorganisms [73,74]. However, the properties of ‘disease-suppressive soils’ can be exploited in the conservation biological control of soil-borne plant diseases. Some studies have deciphered the microbiological basis for the disease-suppressive effect [75,76,77], and a good understanding of these mechanisms can foster the development of directed approaches to the control of soil-borne disease [74,78,79]. Resident microorganisms with pest-control ability constitute a source of strains that can be developed into commercially available pest-control products [3,80,81].

### 3.2. Enhancing the Numbers or Activity of ‘Biostimulating’ Microorganisms

Crop rotation or intercropping with legumes can increase populations of symbiotic root-nodule, nitrogen-fixing bacteria, and shape soil microbial communities [82,83,84], in turn leading to enhanced growth, health, and quality of crops. Similar to the stimulation of resident microorganisms for conservation biological control, ascribing improved plant growth and health induced by a crop management measure to a precise microbiological mechanism according to either the definition for biostimulation or for pest control can be challenging. Thus, in the case of measures that strengthen a plant by improving nutrient availability and acquisition (categorized as biostimulation), it is possible that effects arising from the stimulation of antagonists of plant pathogens (biological control) also contribute to the enhanced growth and health of the plant.

### 3.3. Translocation of Microorganisms to New Locations

In the context of ‘resident’ microorganisms, it should be borne in mind that microorganisms and microbial communities may be transported between locations on a global scale, as a result of the movement of matter and organisms by both abiotic (e.g., weather conditions and water movements) and biotic (e.g., with moving animals) processes. On a local scale, various arthropods, for example pollinating insects, can transfer symbiotic or hitch-hiking fungi, bacteria, or viruses to plants. While many of these transported microorganisms are not likely to have any notable effects on plant growth or health, some can have a positive or negative impact [85,86,87,88]. We are not aware of any legislation that would be directly applicable to stimulating the spontaneous movement of beneficial microorganisms associated with resident arthropods to plants. However, in this context, there are two relevant points:

(i) Several EU countries have national legislations requiring authorization of invertebrate ‘natural enemies’ used in augmentative biological control (especially in greenhouses) of plant pests [89]. Guidelines for the evaluation of invertebrate biological control organisms state that the presence of any microorganisms carried (unintentionally) by animals should be evaluated [90].

(ii) Reared insects can be used as vectors for microbial PPPs [91]. In this case the microorganism is categorized as an active agent of a PPP, and thus must be authorized, while the insect’s role is restricted to being a vector for the delivery of the microorganism to the site of activity.

Additionally, the composition of microbial communities in cropping systems can change in response to management measures, although usually an effect on microbial diversity is not the primary aim of such measures. For instance, changes in land use or application of organic fertilizers can reshape microbial metabolic processes and the community composition [92,93].

### 3.4. Is Targeted Stimulation of Resident Microorganisms Regulated?

We are not aware of any regulatory frameworks that directly target stimulation of resident plant-beneficial microorganisms by management measures, unless the measures themselves include any regulated actions or products. In contrast to the risk-based regulation of augmentation with domesticated microorganisms, the resident microbial biodiversity exerting a level of pest control in agricultural systems is not viewed as potentially harmful to the environment, but as an ecosystem service (to humans) worthy of protection [2]. We concur that this appears to be a sound approach and have found no scientific support for the introduction of any regulatory provisions to actively steer microbial functions with management measures.

## 4. Assessing the Safety of Plant-Beneficial Microorganisms

### 4.1. Hazards and Potential Risks of Microorganisms

The intrinsic biological and ecological properties of a microorganism are always the cornerstones for determining its safety and must be well understood. The hazards and potential risks of microorganisms are briefly summarized below, however, see Cook et al. [94] and Sundh et al. [18] for more detailed analyses.

With respect to humans, microorganisms can potentially be pathogenic and cause infections. Another fundamental hazard is the potential production of toxin(s). Some microorganisms can produce sensitizing substances that can contribute to allergies. For domesticated plant-beneficial microorganisms, potential risks to the staff involved in pre-application production and handling of the organism, and the product end-users, may need to be considered, as well as any potential unintentional exposure of ‘bystanders’ in the cropping system, and during marketing of the crop or final food consumption. For bacteria, it must be ensured that they do not carry transferable genes encoding antibiotic resistance that could exacerbate the problem of increasing resistance to antimicrobials of human or veterinary importance.

Regarding effects on non-target organisms (NTO) in the environment (including agricultural production systems), as for humans there may be a risk of potential pathogenicity or production of toxins. Such effects could lead to changes in the species composition of different groups of organisms and thus also to shifts in the functional properties of a system. Sensitization and spread of antibiotic resistance are less relevant as hazards for NTOs in the environment.

The identification of a new microorganism and the evaluation of its effectiveness and safety should be performed at strain level. Two aspects that are specific to particular microbial strains, and strongly connected to their safety, are the potential capacity to produce toxins and presence of transferable genes coding for antibiotic resistance determinants [95].

### 4.2. Do Potential Risks Differ Depending on Intended Activity?

Any inherent hazards of a particular, wild-type microbial strain are the same, independent of the intended type of beneficial effect. The methods of production, post-fermentation processing, formulation (drying techniques etc.), and application (seed inoculation, soil application, spraying etc.) of plant-beneficial microorganisms in the cropping system are also similar regardless of the claimed effect. Consequently, for many microorganisms any unintended effects on humans or the environment will be similar irrespective of whether the organism is claimed to have a pest control or biostimulating effect. In contrast, the methods of, e.g., manufacture or application of a microorganism can be exclusive for certain taxonomic groups or the environmental compartment and site of activity. For instance, potential risk due to exposure of humans or NTOs in the environment may differ between applications as seed coating and foliar spraying.

Additionally, it is clear that all organisms are not equally likely to be of interest to all functions and applications. For instance, entomo- or phytopathogenic microorganisms (bacteria, fungi, and viruses) that are used in the biological control of pest insects and weeds, respectively, are less likely to be used exclusively as MBSs. The target host range and potential risk for effects on NTOs are particularly critical to evaluate when pathogens are used for pest control, but are usually of less concern for microorganisms used to control pests by other mechanisms.

In short, the potential risk when a domesticated microorganism is applied in cropping systems depends mainly on the organism’s fundamental biological properties and hazards, and to a lesser extent the claimed activity. Thus, we argue that, from a risk perspective, there is little scientific support for having two separate frameworks for regulating microbial products for pest or disease control versus plant biostimulation.

## 5. Towards a More Harmonized Risk Perspective and Regulations

### 5.1. One Microorganism Can Contribute to Both Pest Control and Growth Stimulation

In the natural environment, one specific microorganism performs many different activities and transformations and, hence, provides several functions [96,97,98] and ecosystem services. A relevant example is a specific rhizosphere bacterium that can confer benefits to a plant with the production of both phytohormones (and thus be categorized as a biostimulant) and substances with antibiosis effects on phytopathogens (categorized as a plant protection product) [96,99]. Additionally, there is no clear taxonomic division between microorganisms that have been used for plant disease control or for biostimulation, and strains belonging to, for example, the big bacterial genuses *Bacillus* and *Pseudomonas* and the fungal genus *Trichoderma* can be useful in both areas of application [100,101]. Thus, a similarly wide range of microorganisms from groups with divergent properties are of interest in both areas.

### 5.2. Is There Motivation for a Multitude of Regulatory Approaches?

Regardless of the claimed effect, the basis for a satisfactory safety evaluation is an in-depth understanding of the biological and ecological properties of the evaluated microorganism (and its functions, and targets in the case of microbial pest control). Thus, the scientific basis for evaluating beneficial microorganisms needs to have both the depth required for evaluating particular microbial strains, and the breadth required for understanding the contrasting properties and hazards of diverse groups of microorganisms. We argue that the current policy of decentralized, dispersed regulatory/registration procedures for beneficial microorganisms in cropping systems across several different frameworks and regulatory institutions results in suboptimal evaluation and inefficient use of common resources.

### 5.3. Possible Scenarios for the Revision of Regulatory Frameworks

Several additional points are relevant to a discussion of possible scenarios for regulatory reform regarding plant-beneficial microorganisms.

(i) A (still) growing body of evidence suggests that the PPP framework is not well suited to the regulation of microorganisms, partly because of inbuilt traditions inherited from the authorization of chemical pesticides. The regulatory procedure for MCAs does not equate well with the hazards and risks of microorganisms, and regulations have hampered the implementation of MCA products in the EU (see Section 2.1.2). We believe that the regulatory imbalance regarding microorganisms in crop production is largely caused by overly strict regulation of MCAs, rather than by insufficient attention to potential safety concerns surrounding the use of microorganisms in other application areas (see Table 2 in Sundh and Eilenberg [7] for a fuller overview of regulatory approaches within different sectors).
(ii) We conclude that the example of authorizing MCAs within the PPP regulatory framework reflects the general disadvantage of placing the assessment of microorganisms under established, large, frameworks that were originally designed for other types of agents and/or substances. This suggests that introducing new regulatory systems that are dedicated to microorganisms, or alternatively mandating the assessment of microorganisms to dedicated units and personnel within current sectorial frameworks, would lead to more relevant and efficient authorization processes.
(iii) Efforts to improve regulatory frameworks for plant-beneficial microorganisms can consider either revisions within the current frameworks in the (relatively) short term, or more over-arching revisions in the longer term. Significant experience has been gained in authorizing MCAs as PPPs (began ca 30 years ago), and work to rationalize the process is ongoing within the Farm to Fork initiative of the EU Commission. Regarding the MBSs, the success of the first common EU framework, Regulation 2019/1009 [11], cannot yet be evaluated because the legislation has not come into force. Our tentative scenarios therefore focus on more over-arching, extensive legislation changes. As changing any legislation is a long-term undertaking, this focus should not be taken to imply that ongoing work to improve evaluations within the current regulatory frameworks is less important. Intensive work is necessary to adapt the current legislation and evaluation processes as far as possible, to make them fit-for-purpose regarding microorganisms and their corresponding products.

Four possible scenarios for the development of new regulatory frameworks for the use of plant-beneficial microorganisms in the EU, along with potential advantages and disadvantages, are presented in Table 2. However, any options for over-arching reforms of the regulatory frameworks need further, careful investigation. As well as issues regarding data requirements, principles for evaluation and the need for adequate expertise, any new framework(s) must be organized under an appropriate regulatory institution(s). Thus, these preliminary scenarios should be viewed as ideas for further discussion of reforms that could be implemented in the relatively long term.

## 6. Conclusions and Recommendations

- We conclude that, from the perspective of potential risks, there is little scientific support for separate, contradictory regulatory systems within the EU for evaluating augmentative approaches to using plant-beneficial microorganisms that act via control of plant pests or other modes of action.
- Spreading the regulation of plant-beneficial microorganisms, based on the intended effect and product claims, across different frameworks that were not originally developed for microbiological products, can lead to inefficient evaluations, with a weak basis in microbiological science, and a waste of common societal resources.
- Revised regulatory framework(s) for plant-beneficial microorganisms should ensure that the organisms and products are evaluated within a microbiological and ecological context. Authorizations/registrations need to view the wider perspective of the benefits (as well as any hazards and safety issues) of using microorganisms in different sectors, e.g., food, feed, and biotechnology.
- Our review indicates that a higher level of harmonization of the regulatory policies for plant-beneficial microorganisms in the EU would lead to their more efficient implementation in crop production. Additionally, we conclude that the introduction of regulatory processes and personnel that are dedicated to microorganisms and their corresponding products would improve both the relevance and efficiency of the authorization processes.
- Finally, we recommend further investigations into the advantages and disadvantages of different scenarios for over-arching reformation of the regulations. Such studies are essential before any well-reasoned harmonization of the regulatory policy for plant-beneficial microorganisms can be realized.

## Figures and Tables

**Table 1:** An overview of the current regulatory approaches to the augmentative use of beneficial microorganisms in cropping systems. MO = microorganism, DR = data requirement, PPP = plant protection product, MCA = microbial control agent, MBS = microbial biostimulant, EFSA = European Food Safety Authority.

Framework	Basic Features	Definitions	Legislation	Comments
Microbial control agents				
EU PPP regulations	Authorization in two steps: (i) Approval of the active substance at the EU level; (ii) National authorization of formulated products Two categories of active ‘substances’: (i) chemicals; (ii) microorganisms PPP regulation covers MOs that induce a plant’s tolerance to *biotic* stress, e.g., pests and diseases	DRs: “The term ‘micro-organism’ applies to, but is not limited to, bacteria, fungi, protozoa, viruses and viroids.”	Regulation 1107/2009 for PPPs [24] Directive 2001/36 containing DRs for microbial PPPs [25], re-published in Regulation 283/2013) [26] Directive 2005/25 containing uniform evaluation principles for microbial PPPs [27], re-published in Regulation 546/2011) [28]	Chemical PPPs include semiochemicals and other biologically produced compounds, including extracts from plants or other organisms
EU biocide regulations	Specific DRs for MOs, as for PPPs	Microbial biocides are principally all MCAs that protect items other than plants or plant produce	Regulation EC 528/2012 on authorization of biocides [29]	The DRs for MOs in biocides are given in Annex II in 528/2012. A guidance document on microbial biocides has been developed by European Chemicals Agency (ECHA) [30]
National regulations for ‘microbial pesticides’ outside the EU	Categorization and evaluation procedures for what EU defines as microbial PPPs vary between countries		International overview is provided by Kabaluk et al., 2010 [31]	DRs for ‘microbial pesticides’ in some legislation comparable to the EU have been published by: USA Environmental Protection Agency [32], Australian Pesticides and Veterinary Medicines Authority [33], and Government of Canada [34]
**Microbial biostimulants**				
Current national regulations for biostimulants in the EU and elsewhere	Include e.g., microbial ‘plant strengtheners’, plant growth-promoting rhizobacteria, diazotrophic bacteria in legumes	Differ among countries	Some countries have authorization systems for plant biostimulating MOs that are not classified as PPPs, while others have not	Overviews of the regulations and DRs in different countries are provided by Traon et al., 2014 [35] and Caradonia et al., 2018 [36]
Forthcoming new EU fertilizer regulations (see Appendix A for more details)	Cover biostimulants, i.e., substances or MOs applied in crop production to improve nutrient availability or use, tolerance to *abiotic* stress, or crop quality traits	“‘Plant biostimulant’ means a product stimulating plant nutrition processes independently of the product’s nutrient content with the sole aim of improving one or more of the following characteristics of the plant or the plant rhizosphere: (a) nutrient use efficiency; (b) tolerance to abiotic stress; (c) quality traits; (d) availability of confined nutrients in soil or rhizosphere.”	Regulation (EU) 2019/1009 on EU fertilizer products [23]	The new regulations stipulate that when a MO and its corresponding product fulfil two criteria, it can be added to a positive list, obtain a CE marking and be sold across the EU. Formal pre-market authorization will not be required. MBSs that have not obtained a CE marking according to the new fertilizer regulations can still be marketed in the EU, provided that any national regulations are respected
**Other biotechnical uses**				
EU legislation for environmental applications of MOs (other than as agents of pest control or plant biostimulation), e.g., bioremediation	No common EU framework in place, but national legislation can be applicable		As an example, Sweden has an Environmental Act containing provisions for ‘Biotechnical organisms’. These state that anyone importing and/or using a biotechnical organism is responsible for operational safety. Since authorization is not required, there are no DRs	

**Table 2:** Tentative scenarios for the development of new regulatory frameworks for the use of plant-beneficial microorganisms within the European Union, with potential advantages (+) and disadvantages (−). MCA = microbial control agent, MBS = microbial biostimulant, PPP = plant protection product.

Scenario	Advantages	Disadvantages
(i) Development of a new, dedicated, framework for all plant-beneficial microorganisms, including at least MCAs and MBSs	+ Evaluations and regulatory institutions dedicated to microorganisms + Stronger scientific basis (only microorganisms) + Centralization, facilitating the establishment of a center of expertise	− Regulatory separation of microorganisms from non-living compounds/substances of biological origin − Difficult to identify a suitable regulatory institution(s)
(ii) Development of a new, dedicated, framework for plant-beneficial microorganisms and ‘nature-based substances’, in products claiming either pest control or biostimulation	+ Stronger scientific basis (only ‘nature-based’ agents) + No separation of microorganisms from compounds/substances of biological origin + Centralization, facilitating the establishment of a center of expertise	− Regulatory separation of non-living compounds of biological origin from non-living compounds of synthetic origin − No dedicated evaluation of microorganisms − Difficult to identify a suitable regulatory institution(s)
(iii) (Only pest control) development of a new, dedicated, EU framework for all microorganisms to be used for biological control, i.e., both current PPPs and biocides	+ Stronger scientific basis (only microorganisms) + Centralization, facilitating the establishment of a center of expertise	− (Continued) regulatory separation of different categories of plant-beneficial microorganisms − Difficult to identify a suitable regulatory institution(s) (?)
(iv) (Only pest control) semi-detachment of MCAs from other PPPs within new, separate, subunits of the responsible regulatory institutions, with dedicated personnel	+ Evaluations and institutions dedicated to microorganisms + Stronger scientific basis (only microorganisms) + No need for a new suitable regulatory institution(s)	− (Continued) regulatory separation of different categories of plant-beneficial microorganisms − Regulatory separation of microorganisms from non-living compounds/substances of biological origin

## Data Availability

Not applicable.

## Appendix A

Requirements for the registration of a microbial plant biostimulant (MBS) according to EU Regulation 2019/1009 [23] for fertilizers (adapted from Sundh and Eilenberg [19]).

As a starting-point, Regulation 2019/1009 states that an MBS product can contain microorganisms that:

‘– have undergone no other processing than drying or freeze-drying; and – are listed in the following table:

*Azotobacter* spp.

Mycorrhizal fungi

*Rhizobium* spp.

*Azospirillum* spp.’

Microorganisms that fulfil these two criteria relating to drying methodology and taxonomy can be added to the positive list (introductory point 59 of the Regulation). A product containing a microorganism on the list is considered to comply with the Regulation and can obtain CE marking, meaning it can be moved freely across the internal market of the entire European Union (EU). The list is expected to be updated with additional microorganisms and the Regulation states that ‘Specific rules governing the affixing of the CE marking in the case of EU fertilizing products should be laid down’ (introductory point 41).

To add a new MBS, that does not fulfill the two criteria given above, to the positive list, the following information must be submitted:

‘(a) Name of the micro-organism; (b) taxonomic classification of the micro-organism: genus, species, strain and procurement method; (c) scientific literature reporting about safe production, conservation and use of the micro-organism; (d) taxonomic relation to micro-organism species fulfilling the requirements for a Qualified Presumption of Safety as established by the European Food Safety Authority; (e) information on the production process, including, where relevant, processing methods such as spray drying, fluid-bed drying, static drying, centrifugation, deactivation by heat, filtration and grinding; (f) information on the identity and residue levels of residual intermediates, toxins or microbial metabolites in the component material; and (g) natural occurrence, survival and mobility in the environment.’

All plant biostimulants registered according to the new EU regulations must fulfil the requirements regarding the maximum content of heavy metals and contaminating microorganisms, e.g., common human bacterial pathogens such as *Salmonella* spp. and *Staphylococcus aureus*.
